# P-309. Characteristics of Latine/x Newly Diagnosed with HIV in San Francisco 2021-2023

**DOI:** 10.1093/ofid/ofaf695.528

**Published:** 2026-01-11

**Authors:** Julieta L Rodriguez, Rachel Abbott, Lisa Geronimo, Noel Sergio Leon, Fernanda Amaya, Shalom Bandi, Susan P Buchbinder, Trang Nguyen, Dave Graham-Squire, Diane Havlir, Stephanie E Cohen, Carina Marquez

**Affiliations:** University of California San Francisco, San Francisco, CA; University of California, San Francisco, San Francisco, California; University of California San Francisco, San Francisco, CA; UCSF, San Francisco, California; University of California San Francisco, San Francisco, CA; Latino Task Force, San Francisco, California; San Francisco Department of Public Health, San Francisco, California; San Francisco Dept of Public Health, San Francisco, California; UCSF, San Francisco, California; UCSF, San Francisco, California; San Francisco Department of Public Health, San Francisco, California; University of California, San Francisco, San Francisco, California

## Abstract

**Background:**

The Latine/x population is disproportionately affected by HIV. Prompted by a rise in the number of new HIV diagnoses among Latine/x in San Francisco in 2022 and a request from San Francisco’s Getting to Zero Latine Working Group, we sought to assess socio-demographic characteristics, PrEP awareness, and prior PrEP use among Latine/x newly diagnosed with HIV in San Francisco from 2021-2023. The overall goal of this analysis was to inform HIV prevention and testing strategies in the Latine/x community.

**Figure:**
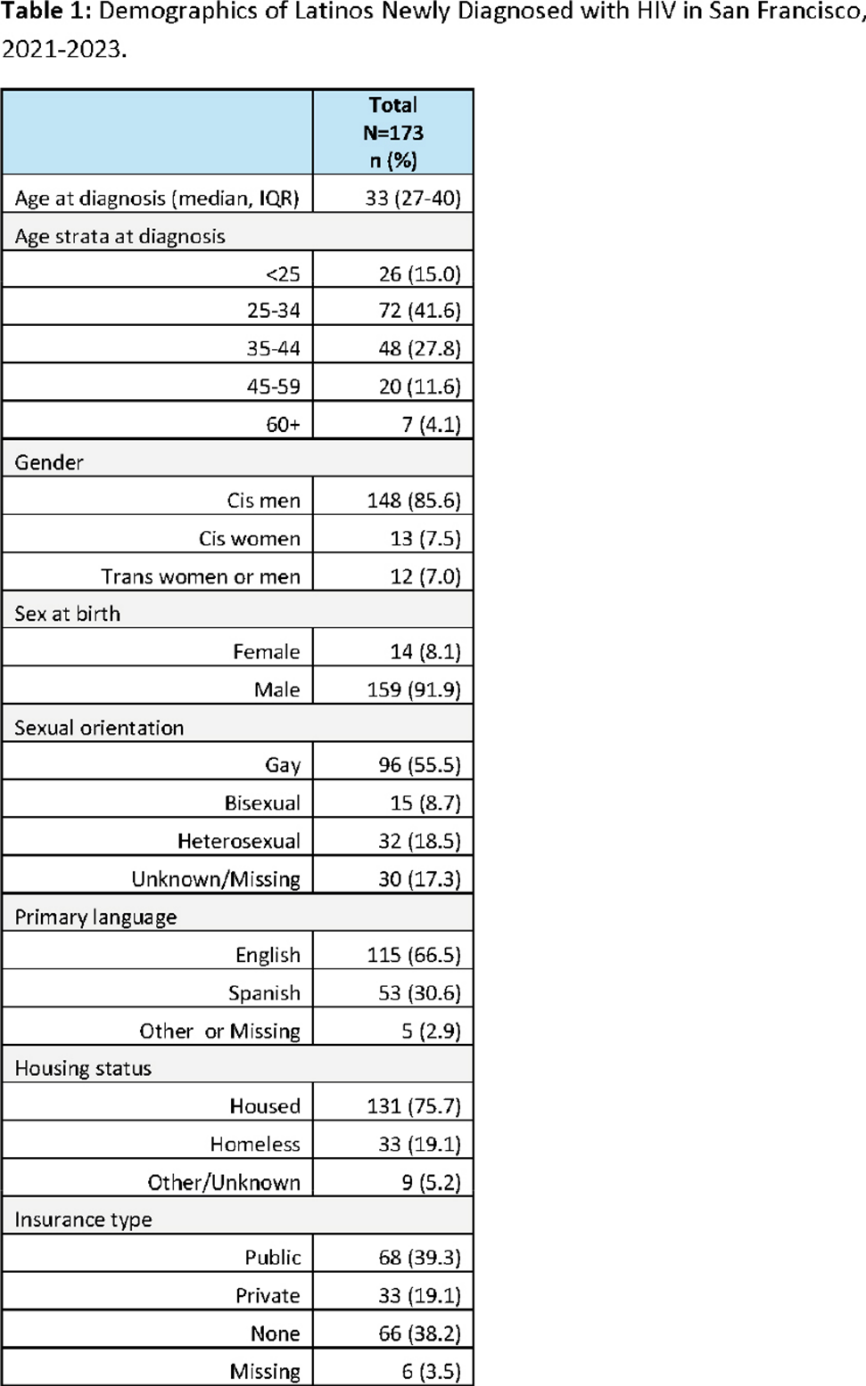

**Figure:**
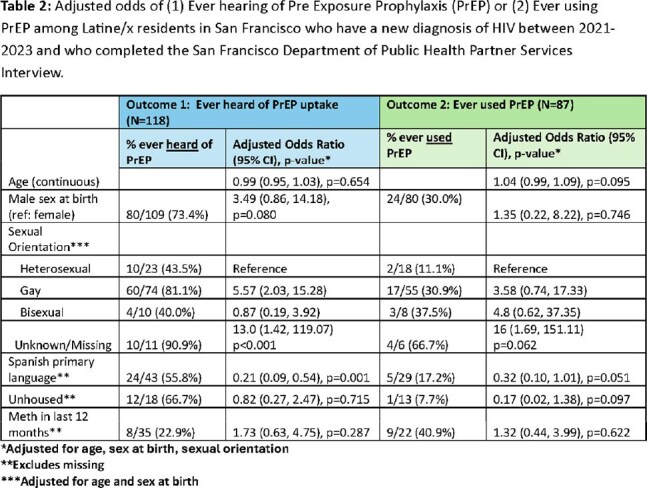

**Methods:**

This is a cross-sectional descriptive study of Latine/x adult San Francisco residents who were diagnosed with HIV from 2021-2023. We analyzed demographic and behavioral data abstracted from the electronic HIV/AIDS reporting system (eHARS), STI surveillance registry (ISCHTR) and San Francisco Department of Public Health’s electronic health record. In the subset of individuals who participated in a partner services interview (provides confidential partner notification, counseling and linkage to healthcare), we used logistic regression to identify predictors of PrEP awareness and prior use, adjusting for age at diagnosis, sex at birth, and sexual orientation.

**Results:**

Between 2021-2023, there were 173 Latine/x San Francisco residents who were newly diagnosed with HIV. The median age is 33 years (IQR 27-40), 86% were cis-men, 56% identify as gay, 31% speak Spanish as their primary language, 19% are unhoused, and 38% are uninsured (Table 1). Among the people who completed a partner services interview and who answered questions on PrEP, 71% (84/118) report having heard of PrEP and 30% (26/87) reported prior PrEP use. The odds of ever hearing of PrEP were lower among people whose primary language is Spanish (aOR 0.2, 95% CI: 0.09-0.54, p< 0.01) compared to English. For ‘PrEP use ever’, odds were lower for people whose primary language is Spanish compared to English (aOR: 0.32, 95% CI: 0.10- 1.01, p=0.051).

**Conclusion:**

Among Latine/x San Francisco residents newly diagnosed with HIV, PrEP use and awareness is lower among those whose primary language is Spanish. These data highlight opportunities to increase Spanish language outreach low-barrier sexual health services and PrEP, including long-acting PrEP.

**Disclosures:**

Susan P. Buchbinder, MD, Aspire Scientific: Medical writing support|Gilead Sciences, Inc.: Grant/Research Support|Gilead Sciences, Inc.: Medical writing support|Merck: Grant/Research Support|ViiV Healthcare: Grant/Research Support Diane Havlir, MD, VIiV: Grant/Research Support Stephanie E Cohen, MD, Cepheid: Grant/Research Support|Hologic: Grant/Research Support|Roche Molecular Systems, Inc: Grant/Research Support

